# Protocol Biopsy After Kidney Transplant: Clinical Application and Efficacy to Detect Allograft Rejection

**DOI:** 10.7759/cureus.34505

**Published:** 2023-02-01

**Authors:** Mahmoudreza Moein, Sarah Papa, Noelle Ortiz, Reza Saidi

**Affiliations:** 1 Transplantation Services, Upstate University Hospital, Syracuse, USA

**Keywords:** dd-cfdna testing, outcomes, protocol biopsy, rejection, kidney transplant

## Abstract

Background

Kidney transplant rejection is a major cause of graft dysfunction and failure. In recent years, there has been increased interest in renal allograft protocol biopsies to allow earlier detection of acute or chronic graft dysfunction or rejection to improve long-term graft survival and reduce graft failure. This study aimed to determine if renal allograft protocol biopsies performed within the first 12 months after transplantation help detect subclinical graft dysfunction or rejection.

Methods

We performed a retrospective analysis utilizing SUNY Upstate University Hospital data from January 2016 to March 2022 to assess transplant outcomes and biopsies. The study population was divided into two subgroups: non-protocol biopsies and protocol biopsies within the 12 months post-transplant.

Results

A total of 332 patients met our inclusion criteria and were included in the study. Patients were divided into two subgroups: 135 patients (40.6%) in the protocol biopsy group and 197 patients (59.4%) with non-protocol indication biopsies during the first year after the transplant. The overall number of rejection episodes reported was eight episodes (4.6%) in the protocol biopsy group and 56 episodes (18.3%) in the non-protocol indication biopsy group, which was significantly higher in the non-protocol biopsy group (P=0.001). Antibody-mediated rejection (ABMR) and T-cell-mediated rejection (TCMR) diagnoses were significantly higher in the non-protocol biopsy group (P=0.03 and P=0.03, respectively). We also mentioned a trend in terms of mixed antibody-mediated rejection and T-cell-mediated rejection diagnosis (P=0.07). One year after the rejection, the mean glomerular filtration rate (GFR) was 56.78 mL/min/1.73m^2^ in the protocol biopsy group and 49.14 mL/min/1.73m^2^ in the non-protocol indication biopsy group, and there was no significant difference anymore (P=0.11). The patient survival rate was not significantly higher in the protocol biopsy group compared to the non-protocol indication biopsy group (P=0.42).

Conclusion

This study suggests that performing protocol biopsies does not significantly benefit rejection rates, graft survival, or renal function within the first 12 months post-transplant. Given these results and the small but non-zero risk of complications associated with protocol biopsies, they should be reserved for those patients at high risk of rejection. It may be more feasible and beneficial to utilize less invasive tests, such as DSA and dd-cfDNA testing, for early diagnosis of a rejection episode.

## Introduction

Kidney transplantation remains the preferred treatment for end-stage renal disease (ESRD) patients. Transplantation should be discussed with all patients with chronic kidney disease (CKD) [1]. Kidney transplantation is associated with decreased morbidity and mortality and increased quality of life compared to chronic dialysis treatment [2,3]. Furthermore, a longer waiting time on dialysis correlates with reduced graft and patient survival, making early kidney transplants essential for improving outcomes [4,5]. In the United States, in 2019, 24,502 kidney transplants were performed, a record high, and a 10.2% increase over the previous year [6].

Kidney transplant rejection is a major cause of graft dysfunction and failure. Antibody-mediated rejection (ABMR) and T-cell-mediated rejection (TCMR) are the most important causes of graft rejection [7]. Other causes of graft rejection include vascular thrombosis, urinary obstructions and strictures, and calcineurin inhibitor nephrotoxicity [8,9]. Rejection is indicated by decreased kidney function, seen as increased serum creatinine. However, subclinical graft rejection is possible, in which damage is occurring to the graft, but the renal function is not yet affected [10]. This can only be diagnosed with a renal biopsy. A renal allograft biopsy is considered the gold standard for diagnosing kidney transplant rejection and should be pursued when there is decreased graft function, often co-occurring with proteinuria and hypertension, which is concerning for rejection [11]. Biopsy specimens are evaluated using the Banff classification. Under this system, T-cell-mediated rejection is diagnosed as acute when tubulitis and vasculitis are present and chronic when atrophic tubules and interstitial fibrosis are present. In contrast, antibody-mediated rejection is classified as acute when there is active tissue injury, microvascular inflammation, positive C4d staining in the peritubular capillaries, and positive donor-specific antibodies [12,13].

In recent years, there has been increased interest in renal allograft protocol biopsies to allow earlier detection of acute or chronic graft dysfunction or rejection to improve long-term graft survival and reduce graft failure. Protocol biopsies are biopsies performed at specific intervals after kidney transplants, such as three months, six months, and one year, regardless of graft function. Performing a protocol biopsy immediately after transplantation allows for the establishment of baseline graft conditions, or if performed one hour after transplant, provides for the evaluation of early immune reaction and reperfusion injuries [14]. Retrospective studies of three-month and two-year post-transplant protocol biopsies have shown improved death-censored graft survival in the biopsy group vs. the non-biopsy group at either five- or seven-year post-transplant. The authors have attributed it to earlier detection and treatment of subclinical rejection and prevention of late rejection [15,16]. However, the overall rejection rate and the death-censored graft survival rate have been found to be similar between the two groups 10 years after a two-year post-transplant protocol biopsy [16]. Despite the proposed benefits of protocol biopsies in improving graft survival, questions remain about their usefulness [11].

Our study aimed to determine if renal allograft protocol biopsies performed within the first 12 months after transplantation help detect subclinical graft dysfunction or rejection. Furthermore, we aimed to determine if implementing protocol biopsies leads to patient management changes that improve rejection rates and graft survival.

## Materials and methods

Study design 

We performed a retrospective analysis utilizing SUNY Upstate University Hospital data from January 2016 to March 2022 to assess transplant outcomes and biopsies. The data were collected from the electronic medical record system (EPIC). Inclusion required patients who received a renal transplant and adult recipients (> 16 years old). Simultaneous kidney-pancreas, other multiorgan transplant recipients, pediatrics (<16 years old), and mortality in 30 days were excluded. A renal biopsy was done at the center by the transplant surgeon, and the result was reported by the pathologist at SUNY Upstate Medical University’s pathology department. The study population was divided into two subgroups: non-protocol biopsies and protocol biopsies within the 12 months post-transplant. The non-protocol biopsy cases were patients who presented with an alarm sign for possible rejection, such as an increase in serum creatinine above the patient’s baseline value, unexplained fever, edema, hypertension, oliguria, anuria, and proteinuria unrelated to glomerulonephritis with the first 12 months post-kidney transplant. Protocol biopsies were within the first 12 months post-transplant in patients with stable serum creatinine and without any clinical manifestation suspected of kidney rejection. The baseline characteristics of the groups of interest were compared. The graft rejection types were reported by the pathologist as antibody-mediated rejection (ABMR), T-cell mediated rejection (TCMR), borderline rejection (BR-L), and mixed rejection with both antibody-mediated and T-cell mediated rejection at the same time. The Banff 2013 rejection criteria and components were implanted to assess and compare the histopathological features of rejection in each group as the protocol [17].

Outcome definitions

The primary objective was to assess the protocol biopsies results, compare the outcomes with the kidney recipients that did not have the protocol biopsies as part of their post-transplant screening, and compare the acute rejection rate and graft failure. The secondary objective was to assess how protocol biopsies could improve the patient and graft survival rates.

Statistical analyses

The primary analyses measured the baseline characteristics in the groups of interest. The t-test and analysis of variance (ANOVA) performed univariate analysis for continuous variables, chi-square test for categorical variables, and Kaplan-Meier curves to assess patient and graft survival rates. Categorical data were summarized as proportions and percentages, and continuous data were summarized as means and standard deviations. A P-value of <0.05 was considered significant, and a P-value of < 0.1 was considered a trend.

## Results

**Figure 1:**
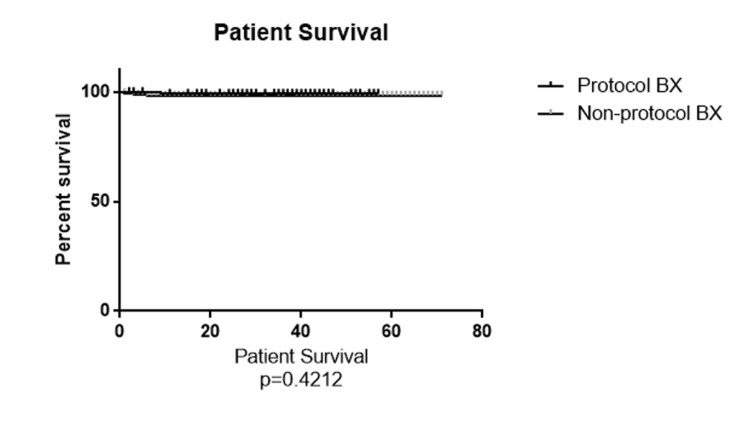
Five-year patient survival rate comparison between the protocol and non-protocol biopsy groups

**Figure 2:**
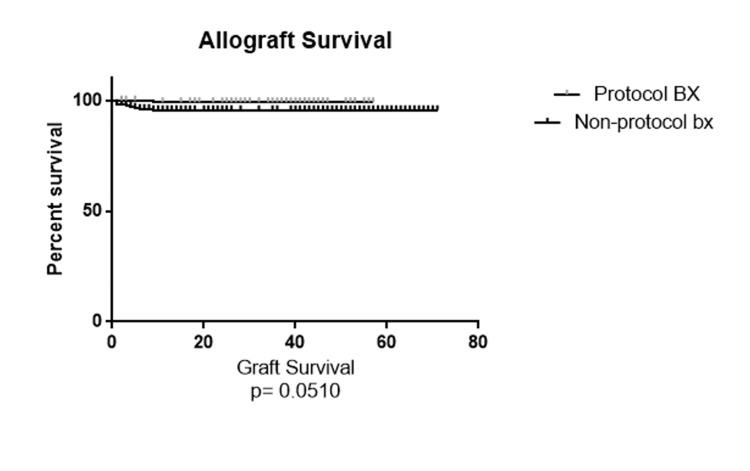
Five-year allograft survival rate comparison between the protocol and non-protocol biopsy groups

A total of 332 patients met our inclusion criteria and were included in the study. Patients were divided into two subgroups: 135 patients (40.6%) in the protocol biopsy group and 197 patients (59.4%) with non-protocol indication biopsies during the first year after the transplant. The recipients' demographic characteristics were compared and summarized in Table 1. The total number of biopsies in the non-protocol indication biopsy group was 306 and 175 for the protocol biopsy group within the first year. The overall number of rejection episodes reported was eight episodes (4.6%) in the protocol biopsy group and 56 episodes (18.3%) in the non-protocol indication biopsy group, which was significantly higher in the non-protocol biopsy group (P=0.001). Table 2 shows the distribution of the rejection type in each subgroup. ABMR and TCMR diagnosis was significantly higher in the non-protocol biopsy group (P=0.03 and P=0.03, respectively). We also mentioned a trend regarding a mixed ABMR and TCMR diagnosis (P=0.07). We also compared the different glomerulonephritis incidents diagnosed in each biopsy group. MPGN was the only glomerulonephritis diagnosed significantly higher in the protocol biopsy group compared to the non-protocol indication biopsy group (P=0.04) (Table 3). We also assessed and compared the histopathological features of the biopsies that showed signs of rejection based on the Banff 2013 kidney rejection classification scores. We found that total inflammation (ti) and peritubular capillaritis (ptc) scores were significantly higher in the non-protocol indication biopsy group (P=0.01 and P=0.05, respectively) compared to the protocol biopsy group. Table 4 summarizes the Banff 2013 histopathology score comparison. In order to assess kidney function, we compared the glomerular filtration rate (GFR) at the time of rejection and one year after the rejection in each group. At the time that rejection was confirmed, as expected based on the meaningful difference between the groups based on the kidney donor profile index (KDPI) and delayed graft function (DGF) rate, the mean GFR was significantly better in the protocol biopsy group and was 60.87 mL/min/1.73m^2^ in the protocol biopsy group and 33.51 mL/min/1.73m^2^ in the non-protocol indication biopsy group. One year after the rejection, the mean GFR was 56.78 mL/min/1.73m^2^ in the protocol biopsy group and 49.14 mL/min/1.73m^2^ in the non-protocol indication biopsy group, and there was no significant difference anymore (P=0.11). After one year, the GFR rate declined non-significantly in the protocol biopsy group. It increased significantly in the non-protocol indication biopsy group. Because of that, the comparison of one-year GFR does not show a significant difference even with the higher KDPI and DGF rate in the non-protocol biopsy group. The five-year patient and graft survival rates are shown in Figure 1 and Figure 2. The patient survival rate was not significantly higher in the protocol biopsy group compared to the non-protocol indication biopsy group (P=0.42), and a trend was mentioned in favor of the allograft survival rate in the protocol biopsy group compared to the non-protocol indication biopsy group, and a trend was noted (P=0.051).

**Table 1:** Recipients’ demographics and comparison between the protocol and the non-protocol biopsy groups PRA: panel reactive antibodies, KDPI: kidney risk profile index, CIT: cold ischemia time, DGF: delayed graft function, BMI: body mass index

Parameter	Protocol Biopsy (n=135)	Non-Protocol indication Biopsy (n=197)			P-value
Recipient age (mean ± SD, years)	50.13±12.55		50.24±15.43	0.94	
PRA (%)	21.20±31.97		25.01±35.73	0.32	
KDPI (%)	37.17±28.83		44.75±28.18	0.01	
CIT (mean ± SD, Hours)	18.46±11.15		19.59±11.98	0.38	
DGF rate (%)	21.5		37.6	0.002	
BMI	29.73±6.29		30.17±7.11	0.55	
Donor type, n (%)/Deceased donors	111 (82.2%)		157 (79.7%)	0.66	
Donor type, n (%)/Living donors	24 (17.8%)		40 (20.3%)	0.66	

**Table 2:** Total number of cases, number of performed biopsies, and distribution of different types of rejection in the protocol and the non-protocol biopsy groups Bx: biopsy, ABMR: antibody-mediated rejection, TCMR: T cell-mediated rejection, BR-L: borderline rejection

Parameter	Protocol Bx	Non-protocol indication Bx	P-value
Total cases	135	197	
Number of Bx	175	306	
Overall rejection, n (%)	8 (4.6)	56 (18.2)	0.001
ABMR, n (%)	2 (1.15)	17 (5.5)	0.03
TCMR, n (%)	2 (1.15)	16 (5.2)	0.03
BR-L, n (%)	4 (2.3)	12 (3.9)	0.34
Mix ABMR and TCMR, n (%)	0	11 (3.6)	0.07

**Table 3:** Golmerulonephropathies were diagnosed after biopsy in the protocol and non-protocol biopsy groups IN: interstitial nephritis, FSGS: focal segmental glomerulosclerosis, MPGN: membranoproliferative glomerulonephritis, IgAN: IgA nephritis

Parameter	Protocol Bx	Non-protocol indication Bx	P-value
IN	5	4	0.23
FSGS	1	3	0.63
MPGN	5	1	0.04
IgAN	1	2	0.91

**Table 4:** The Banff 2013 histopathology score comparison *P-value for the compression between the protocol biopsy group and the non-protocol indication biopsy group ti: total inflammation, IFTA: interstitial fibrosis and tubular atrophy, t: tubulitis, g: glomerulitis, ptc: peritubular capillaritis, i: interstitial inflammation

Banff 2013 parameter	Protocol Bx score	Non-protocol indication Bx score	P-value^*^
ti	1-2	2-3	0.01
ifta	1-2	2-3	0.21
t	0-1	1-2	0.37
g	1-2	0-1	0.31
ptc	0-1	1-2	0.05
i	1-2	1-2	0.58

 

## Discussion

In this retrospective study, we demonstrated no benefit to performing protocol biopsy within the first 12 months post-transplant. Specifically, we found a significant difference between the protocol biopsy group and the non-protocol biopsy group in rejection rate, which was higher in the non-protocol biopsy group. There was also no significant difference in renal function, measured via GFR at 12 months post-rejection diagnosis, even though the GFR was significantly higher in the protocol biopsy group at the time of rejection diagnosis and the non-protocol biopsy group had a significantly higher KDPI and DGF rates. However, at the time of diagnosis of rejection, the protocol biopsy group had lower peritubular capillaritis and total inflammation scores. Finally, we found no significant difference in five-year patient survival rates between the two groups and only a trend in improved five-year graft survival rates favoring the protocol biopsy group. These results conflict with recent studies by Terrec et al. and Chen et al., showing that both three-month and two-year protocol biopsies improve long-term graft survival [15,16]. However, it agrees with a study by Rush et al. that showed no benefit to early protocol biopsies on graft survival or renal function in the first six months post-transplant [17].

Many studies have shown that the prevalence of subclinical rejection is variable but relatively low, with rates as low as 2.6-9% in the first three to six months post-transplant and increased slightly to about 27% at one-year post-transplant [18-20]. There are also low rates of borderline changes, amounting to approximately 1.4-11% of patients at three months post-transplant and 4.8% of patients at one-year post-transplant [18-20]. These rates continue to decrease with the advent of increasingly effective immunosuppressant regimens [18]. The naturally low prevalence of subclinical rejection and borderline changes may explain the low yield rate of the protocol biopsies performed in this study. Furthermore, this low yield rate explains why our results reveal that protocol biopsy does not improve patient and graft survival rates or renal function. Suppose subclinical rejection and borderline rejection both have a low prevalence in kidney transplant patients. In that case, protocol biopsies may not be able to identify enough episodes to make a meaningful difference in clinical management.

Subclinical and borderline rejection are also variable in their course and do not always progress to acute rejection. A study of renal biopsies performed serially over the first 12 months post-transplant showed that in patients demonstrating subclinical rejection, it became persistent in only 35.8% of patients and resolved in all others. If these patients had never had a prior acute rejection episode, the risk of persistent subclinical rejection was only 8.8%. For all cases, persistent subclinical rejection resolved in an average of 0.48 years. Although this study found that subclinical rejection could progress to chronic cell-mediated rejection, it was a relatively rare finding in patients treated with calcineurin inhibitor-based immunosuppression therapy, occurring in only 5.8% of cases [21]. Furthermore, Rush et al. found no benefit to identifying and treating subclinical rejection on either graft survival or renal function in the first six months post-transplant for patients maintained on an immunosuppressive regimen of TAC, MMF, and prednisone [18]. The significance of borderline rejection findings is similarly unclear. Previous studies have shown that patients with borderline changes on protocol biopsies at three months post-transplant do not show the progression of histologic abnormalities at the two years follow-up and show no differences in renal function from those without borderline changes at both the one- and two-year follow-ups [22]. However, other studies have shown that borderline changes are associated with decreased renal function and increased incidence of acute tubulitis [23]. Additionally, a small study has shown that 72% of borderline rejection cases that are not treated with further anti-rejection medication will not progress to acute rejection within the next 40 days [24]. When treated with anti-rejection medications similar to acute rejection episodes, only 43-63% of borderline rejection cases demonstrate a complete response, with 13-28% demonstrating a partial response and 25-30% demonstrating no response [25,26]. With both subclinical and borderline rejection having a high likelihood of resolving without additional management and additional anti-rejection therapy showing no or inconsistent benefit in preventing acute rejection and improving graft survival and renal function, performing protocol biopsies to identify these conditions may not provide any additional benefit to transplant patients, as demonstrated in our study.

When considering whether to include protocol biopsies as part of the standard of care post-transplant, it is essential to consider the risks of the biopsy procedure. While complications from renal biopsies are reported to be very low, there is still a possibility of both major and minor complications. In approximately 1% of cases, significant complications include hemorrhage, macroscopic hematuria causing ureteric obstruction, peritonitis, and graft loss [27-29]. Minor complications in 0.5-7.3% of cases include gross hematuria, perirenal hematomas, arteriovenous fistulas that are asymptomatic, and vasovagal responses [29]. Despite the overall low incidence of these complications, the risks of performing a protocol biopsy may outweigh the benefits given the previously discussed low yield of protocol biopsies and the lack of benefit on patient and graft survival rates and renal function found in this study. Therefore, rather than be considered standard of care, protocol biopsies may be better suited to being reserved for patients at higher risk of rejection, which can be predicted with pretransplant indicators, such as the KDPI and PRA, in whom the potential benefits of protocol biopsies are more likely to outweigh the risks as mentioned earlier.

An additional consideration when deciding whether to include protocol biopsies in the standard of care is the availability of other clinical tests that may provide the same or better predictive results for graft rejection and decreased renal function, especially those that are less invasive. In Loupy et al.'s study of one-year protocol biopsies, they found that all patients with subclinical ABMR had donor-specific antibodies (DSA) at the time of protocol biopsy, with 78% having preexisting DSA and the remaining 22% having de novo DSA, in comparison to only 18.2% of patients who did not have subclinical rejection [20]. Furthermore, newer blood tests are being developed to assess donor-derived cell-free DNA (dd-cfDNA) to predict graft rejection. A study by Bu et al. showed that elevation of dd-cfDNA of 0.5% or more correlates significantly with both clinical and subclinical rejection and that dd-cfDNA values of 0.5% or more are associated with an almost three times greater risk of developing de novo DSA. Differences in dd-cfDNA values were also shown to be able to distinguish if a patient had no rejection, rejection of any type, ABMR, or TCMR. Furthermore, persistently elevated dd-cfDNA is predictive of a 25% decline in GFR within a three-year period [30]. Therefore, tests such as DSA and dd-cfDNA may be better able to predict both graft rejection and decreased renal function than protocol biopsies, without the risk of complications associated with protocol biopsies. 

There are some limitations to this study. The study was conducted retrospectively, so we could not control for confounding variables, and there was a risk of selection bias. This was also a single-center study and needed to be validated with an external population. In addition, the retrospective nature of this study did not allow for uniform treatment of subclinical or borderline rejection cases, instead leaving such decisions to the treating physician's discretion. Perhaps implementing a standardized approach to the management of such patients would reveal more compelling evidence to support the identification and prompt clinical treatment of patients with either subclinical or borderline rejection. Finally, the reporting of Banff grades by pathologists at this institution was not performed consistently among pathology reports for all biopsies, leading to loss of data in some cases that may have been able to expose differences in the severity of rejection episodes between the protocol biopsy and non-protocol biopsy groups.

## Conclusions

In conclusion, this study suggests that performing protocol biopsies does not significantly benefit rejection rates, graft survival, or renal function within the first 12 months post-transplant. Given these results and the small but non-zero risk of complications associated with protocol biopsies, they should be reserved for those patients at high risk of rejection as identified using KDPI or PRA scores. In lieu of using protocol biopsies to detect rejection before it presents clinically, it may be more feasible and beneficial to utilize less invasive tests such as DSA and dd-cfDNA testing.
